# The Origin and Evolution of Chromosomal Reciprocal Translocation in *Quasipaa boulengeri* (Anura, Dicroglossidae)

**DOI:** 10.3389/fgene.2019.01364

**Published:** 2020-01-21

**Authors:** Yun Xia, Xiuyun Yuan, Wei Luo, Siqi Yuan, Xiaomao Zeng

**Affiliations:** ^1^ Chengdu Institute of Biology, Chinese Academy of Sciences, Chengdu, China; ^2^ College of Computer Science, Sichuan University, Chengdu, China; ^3^ University of Chinese Academy of Sciences, Beijing, China; ^4^ College of Bioengineering, Sichuan University of Science & Engineering, Zigong, China

**Keywords:** chromosomal rearrangement, recombination suppression, hybrid unfitness, reciprocal translocation, spiny frog

## Abstract

Chromosomal rearrangements have long fascinated evolutionary biologists for being widely implicated in causing genetic differentiation. Suppressed recombination has been demonstrated in various species with inversion; however, there is controversy over whether such recombination suppression would facilitate divergence in reciprocal translocation with reduced fitness. In this study, we used the spiny frog, *Quasipaa boulengeri*, whose western Sichuan Basin populations exhibit translocation polymorphisms, to test whether the genetic markers on translocated (rearranged) or normal chromosomes have driven this genetic differentiation. We also investigated its overall genetic structure and the possibility of chromosomal fixation. Whole-chromosome painting and genetic structure clustering suggested a single origin of the translocation polymorphisms, and high-throughput sequencing of rearranged chromosomes isolated many markers with known localizations on chromosomes. Using these markers, distinct patterns of gene flow were found between rearranged and normal chromosomes. Genetic differentiation was only found in the translocated chromosomes, not in normal chromosomes or the mitochondrial genome. Hybrid unfitness cannot explain the genetic differentiation, as then the differentiation would be observed throughout the whole genome. Our results suggest that suppressed recombination drives genetic differentiation into a balanced chromosomal polymorphism. Mapping to a reference genome, we found that the region of genetic differentiation covered a wide range of translocated chromosomes, not only in the vicinity of chromosomal breakpoints. Our results imply that the suppressed recombination region could be extended by accumulation of repetitive sequences or capture of alleles that are adapted to the local environment, following the spread and/or fixation of chromosomal rearrangement.

## Introduction

Chromosomal rearrangements (CRs) are widely acknowledged to be an important evolutionary force driving genetic differentiation (Ayala and Coluzzi, 2005). CRs can lead to chromosomal speciation, as well as a balanced chromosomal polymorphism in some cases (Brown and O'Neill, 2010; Faria and Navarro, 2010). Most studies of CRs have been on inversions, and have demonstrated that suppressed recombination facilitates divergence and speciation in various species (Hoffmann and Rieseberg, 2008; Neafsey et al., 2010). However, the forces driving evolutionary changes in other types of rearrangements, such as Robertsonian fusions and reciprocal translocations, have rarely been experimentally explored. As reciprocal translocation could cause meiotic problems and reduce the fitness of chromosomal heterozygotes (White, 1978; King, 1993), it facilitating divergence has been explained by both suppressed recombination and hybrid unfitness (Franchini et al., 2010; Giménez et al., 2013). However, for a balanced chromosomal polymorphism caused by reciprocal translocation, it is unclear which of these may be the major driving force.

It is surprising that a different karyotype can be maintained and spread *via* chromosomal radiation or colonization (Faria and Navarro, 2010). There are two possible outcomes of a new rearrangement: most are maladaptive and thus are quickly eliminated from populations by natural selection; however, in a few instances, individuals may rapidly achieve stable fixation and colonize new niches (Coyne and Orr, 2004). These fixed CR karyotypes or polymorphisms have long fascinated evolutionary biologists due to their rarity. Many previous studies have provided evidence for fixation in small isolated populations, presumably having arisen through genetic drift (White, 1973; Britton-Davidian et al., 2000). Local fixation or radiation of a chromosome polymorphism can also occur through selection (Kirkpatrick and Barton, 2006; Hoffmann and Rieseberg, 2008; Kirkpatrick, 2017). There are numerous documented examples of heterosis and cline associated with an environmental gradient of chromosomal races, which implies that selection acts on the fixation of rearrangements (Butlin and Day, 1989; Butlin, 2005). The selection perspective is also supported by the recent discovery of positive epigenetic architectures and fragile breakpoints in rearrangement regions (Larkin et al., 2009; Brown and O'Neill, 2010). At present, many arguments focus on the effect of rearrangements on reproductive isolation but do not consider how such rearrangements are initially spread through populations (Navarro and Barton, 2003b; Horn et al., 2012). Therefore, investigation of the origin of chromosomal spread and colonization would help our understanding of the forces that maintain a balanced chromosomal polymorphism (Kawakami et al., 2009; Feder et al., 2011; Förster et al., 2013).

**Figure 1:**
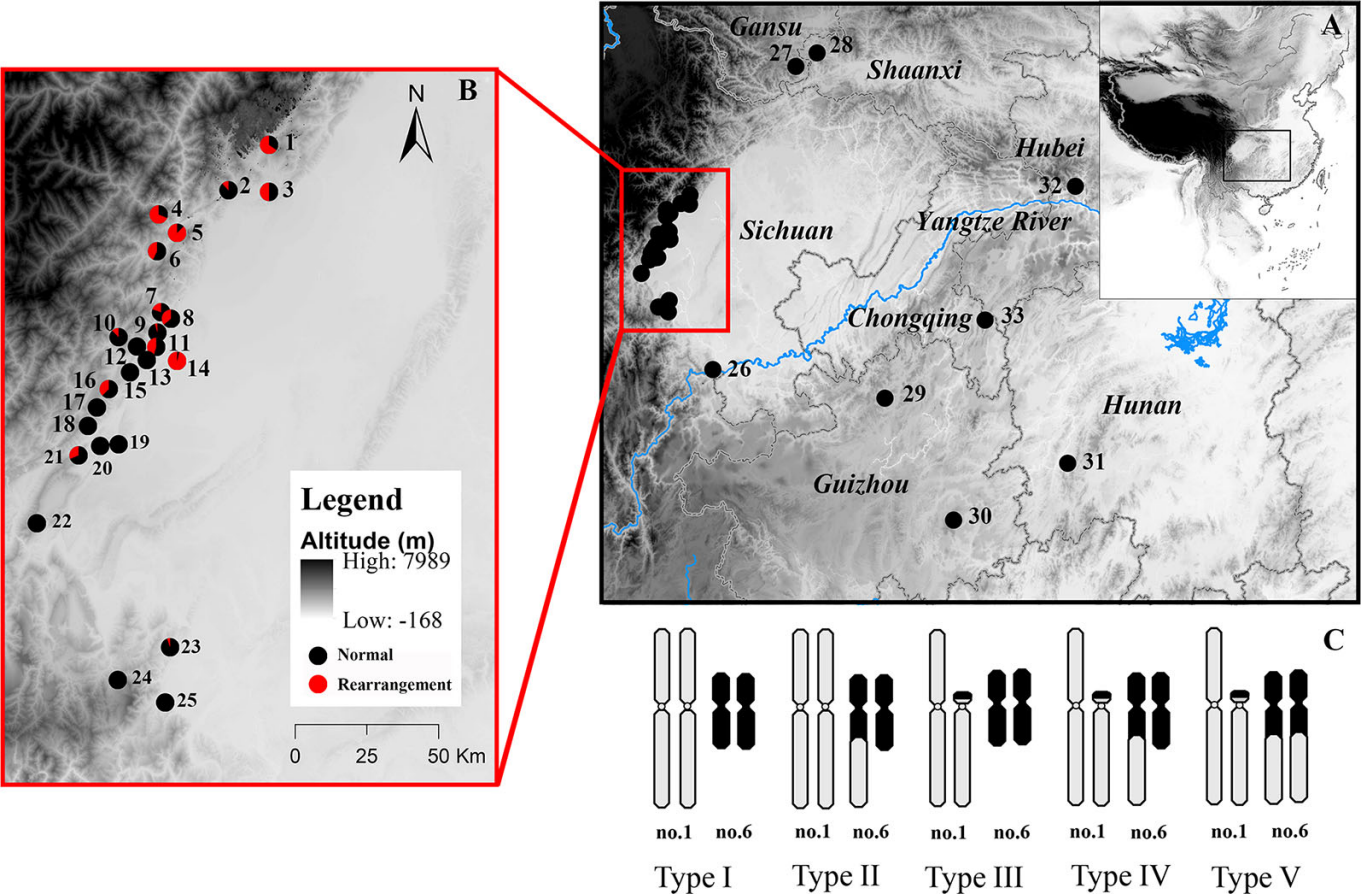
Map of sampling localities and schematic diagram of different karyotypes of *Quasipaa boulengeri*. The site names are the same as in **Table S1**. **(A)** Sampling localities around Sichuan Basin, China. **(B)** Map showing the sites in the western Sichuan Basin, China. **(C)** Five different karyotypes in populations from the western Sichuan Basin. After the translocation rearrangement randomly occurred in a single individual, the translocation heterozygote (type IV) can form a quadrivalent during meiosis and four kinds of gametes, when it mated with a normal individual (type I), individuals with type II and type III were produced (Qing et al., 2012). When individuals with rearranged karyotypes mated with each other, those of nine karyotypes could be produced, while only five kinds of progenies with karyotypes I, II, III, IV, V are observed in natural populations, four kinds of progenies with karyotypes VI, VII, VIII, IX are absent, it implied that fertility and fitness were reduced for the translocated karyotypes. This map is created with ArcGIS (ESRI, http://www.esri.com/software/arcgis).

*Quasipaa boulengeri*, is a frog species widely distributed in low mountainous regions along the edges of Sichuan Basin and nearby areas in southern China, with chromosomal polymorphisms caused by nascent reciprocal translocation but with uniform morphological characteristics (Qing et al., 2012). A total of five karyomorphs have been observed only in western Sichuan Basin populations, including the normal karyotype (type I), translocation heterozygote (type IV), and three hybridized karyotypes (Qing et al., 2012) (types II, III and V; **Figure 1**). Qing et al. (2012) suggested that the reciprocal translocation mutation independently evolved only once in this species. The karyotypic polymorphism in this species has some surprising features. First, a translocation homozygote has not been found in any investigated populations. Three hybridized karyotypes, each of which was thought to have developed adjacent-1 segregation, have only been found in a small number of individuals, probably due to the lethality of translocation homozygosity, suggesting that CRs impair fertility and fitness in this frog (White, 1973; Searle, 1993). Second, no convincing evidence of chromosomal fixation has been found, but the widespread karyotypic variants identified in multiple populations suggest that the CRs are maintained in a balanced state, with chromosomal polymorphisms (Qing et al., 2012). Third, phylogenetic analyses of mitochondrial fragments have shown no divergence between the rearranged western group and the normal eastern and southern groups. This implies that the genetic divergence in these cases does not cover the whole genome but rather may be limited to the translocated (rearranged) chromosomal region. Therefore, it is not clear how a heterozygote with sustained semisterility can disperse among natural populations and maintain a high frequency in some specific populations.

To understand the colonization history of the translocation heterozygotes, we first sought to confirm the single origin of the balanced chromosomal translocation polymorphisms, and investigated the genetic structure of this frog species, especially populations in the western Sichuan Basin, using markers located on both rearranged and normal chromosomes. Then we tested how this translocation has driven population genetic differentiation in this species, by comparing differences in recombination rates between CR and autosomal markers and mapping suppressed recombination markers to the reference genome. Finally, we investigated whether a genetic bottleneck has occurred in the western Sichuan Basin. We discuss how the chromosomal polymorphisms have spread.

## Materials and Methods

### Specimens

A total of 651 *Q. boulengeri* adults were collected from 33 natural populations in China (**Figure 1**, **Table S1**), during the breeding season from 2006 to 2016 (Qing et al., 2012; Xia et al., 2016; Yuan S.Q. et al., 2017). Populations 1–25 were isolated from the western Sichuan Basin, the area where natural chromosomal translocation polymorphisms were found (rearranged areas). Populations 26–33 were from the southern, northern, and eastern areas of their distribution range and had a consistent normal chromosomal karyotype (non-rearranged areas). Genomic DNA was extracted from liver or muscle tissue using the standard proteinase K method (Sambrook and Russell, 2001). All animal work was conducted according to relevant national and international guidelines. All animal care and experimental procedures were approved by the Chengdu Institute of Biology Animal Care and Use Committee (CIB2015003).

### Chromosome Preparation, Microdissection, and Amplification

Mitotic metaphases were prepared from bone marrow using the air-dry method (Schmid et al., 2010). Five *Q. boulengeri* individuals with karyotype I (normal karyotype) were chosen for chromosome isolation. Target chromosomes were mechanically microdissected using the same protocol as described by Yuan X.Y. et al. (2017). Approximately 10 copies of chromosome 1 were microdissected and deposited in a PCR (Polymerase Chain Reaction) tube containing 9 µL ddH_2_O. Specific DNA was amplified *via* single-cell amplification using the GenomePlex^®^ Whole Genome Amplification (WGA) kit (Sigma Chemical Co., USA) following the standard protocol. To avoid any contamination, all instruments and solutions were autoclaved and treated with UV for more than 1 h before use. All manipulations were carried out under aseptic conditions.

### Whole-Chromosome Painting of Different Karyotypes

The five different karyotypes (Types I–V) were used for whole-chromosome painting (WCP). The painting probes were labeled with Dig-11-dUTP (Roche Diagnostics, Germany) *via* whole-genome amplification (WGA: WGA3 kit, Sigma, USA). Painting fluorescence *in situ* hybridization (FISH) was accomplished following the protocol of Yang et al. (1995) with minor modifications. To block highly repetitive DNAs, C_0_t-1 DNA from *Q. boulengeri* was made according to the procedure described by Zwick et al. (1997). The hybridization mix contained 5 ng/μL probe DNA, 40 ng/μL C_0_t-1 DNA, 10% dextran sulphate, and 50% deionized formamide in 2× SSC, denatured at 100°C for 10 min and pre-annealed by incubation at 37°C for 80 min. After hybridization overnight in a wet chamber at 37°C, the probes were detected with anti-digoxigenin-fluorescein fab fragments (Roche, Germany). Chromosomes were counterstained with 4′, 6-diamidino-2-phenylindole (DAPI, Vector). Hybridization signals were observed under a Leica DM2500 fluorescence microscope equipped with a fluorescent lamp and an appropriate filter set.

### High-Throughput Sequencing of Chromosome 1

Library construction and sequencing of chromosomal DNA were performed by Novogene Bioinformatics Technology (Beijing, China). First, the amplified chromosomal DNA was sheared to a size distribution of 300–500 bp. The paired-end (PE) library for chromosomal DNA (5.4 μg) was prepared using the TruSeq DNA PCR-Free Sample Preparation Kit (Illumina, USA), following the protocols for Illumina DNA sample preparation. The library was pooled for sequencing on the Illumina Hiseq2500 (PE250) at Novogene Bioinformatics Technology (Beijing, China).

The raw sequence reads were inspected using FASTQC (www.bioinformatics.babraham.ac.uk/projects/fastqc), and adaptors were trimmed using Trimmomatic (Bolger et al., 2014). In this step, all raw reads with more than 10% missequenced nucleotides (ploy-N) were discarded, as were reads for which more than 50% of the bases had a Q-value ≤20. *De novo* assembly of the cleaned reads for chromosomal DNA sequencing was performed using SOAPdenovo2 (Luo et al., 2012). Read preprocessing and assembly parameters followed each program's guidelines.

### Isolation, Amplification, and Location of Molecular Markers

More than 100 microsatellite loci have been described in *Q. boulengeri* (Xia et al., 2013; Yuan S. Q. et al., 2015; Yuan S. Q. et al., 2017; Xia et al., 2018). Three methods were used to identify polymorphic loci in the CRs. Firstly, we mapped polymorphic loci at the chromosomal levels used the same protocol as described by Yuan S. Q. et al. (2017). Following this protocol, we used mechanical microdissection to collect chromosome 1 from metaphase spreads, and then we isolated chromosomal DNA by the single-cell whole-genome amplification technique. If microsatellites loci were amplified successfully in the chromosomal DNA through PCR, it was thought to be located on CRs. Secondly, we put the amplified chromosomal DNA of chromosome 1 into NGS (next-generation sequencing). The polymorphic microsatellite loci published previously were used as queries for a BLASTN v2.3 search against the database of chromosome 1 using default parameters. If microsatellites loci were mapped on chromosome 1 dataset, it was considered to be located on CRs. Thirdly, we also isolated some microsatellite loci from the chromosome 1 NGS data sets. Besides, for these loci from NGS, we still check its accuracy using the first protocol.

In total, we selected 13 microsatellite loci assigned to CRs (rearranged loci) and 11 loci attached to normal chromosomes (normal loci). These 24 markers were amplified in 651 *Q. boulengeri* individuals collected from 33 populations (**Table S1**). Primer details for each locus are listed in **Table S2**. Negative controls (ddH_2_O) were run for all amplifications. The PCR products were genotyped using an ABI-3730xl sequencer (Sangon Biotech Ltd. Co Shanghai, China). We also developed nuclear gene sequence fragments from high-throughput sequencing of rearranged chromosomes. Four nuclear gene fragments (QBR-B11, QBR-C7, QBR-C27, and QBR-D60) located on CRs were chosen after sequence alignment, and amplified in 10 normal and 13 rearranged karyotypic individuals, respectively. Amplified nuclear gene products were visualized on 2% agarose gels stained with ethidium bromide and purified using the TIANgel Midi Purification Kit (TianGen Biotech, Beijing, China). Purified products were cloned using *Escherichia coli* DH5α (TransGen Biotech, Beijing, China) competent cells, with the pMD19-T vector (Takara, Japan) according to the manufacturer's instructions. Positive cloned fragments were sequenced by Sangon Biotech Ltd. Co. (Shanghai, China).

In addition, four fragments from the mitochondrial genome, cytochrome oxidase I (c*ox1*), cytochrome b (*cytb*), 12S ribosomal RNA (*12s*), and 16S ribosomal RNA (*16s*) and a total of 213 specimens were selected for phylogenetic analysis. The information and primer details are shown in **Tables S1** and **S2**. For nuclear gene fragments and mitochondrial fragments, sequence alignments and haplotype identification were performed using MEGA 7.0 (Kumar et al., 2016) and DAMBE (Xia, 2018). Phylogenetic relationships were constructed using MrBayes 3.2 with a 10,000,000 generations and 0.25 burnin metrics (Ronquist et al., 2012), employing all haplotypes along with several outgroup sequences from GenBank, including *Q. exilispinosa* (KF199151.1), *Q. shini* (KF199148.1), *Q. jiulongensis* (KF199149.1), *Q. spinosa* (FJ432700.1), *Q. verrucospinosa* (KF199147.1), and *Q. yei* (KJ842105.1). The best-fit model was estimated using the Bayesian information criterion (BIC) implemented in PartitionFinder v2.1.1 (Lanfear et al., 2016). To observe the reticulation in nuclear markers, the network analysis was performed by Splitstree (Huson and Bryant, 2005).

### Statistical and Cluster Analyses

Two statistical analyses were performed: comparing the geographic areas of rearrangement (western Sichuan Basin) and non-rearranged regions (populations in eastern, southern, and northern areas), and comparing individuals with rearranged karyotypes and normal karyotypes in the western Sichuan Basin. F-statistics analyses, including assessments of genetic differences among individuals within populations (F_IS_), among loci within individuals (F_IT_), among populations within areas (F_SC_), and between rearranged and non-rearranged areas (F_CT_), were performed using Arlequin 3.5 (Excoffier and Lischer, 2010). Linkage disequilibrium, Hardy–Weinberg exact tests and expected heterozygosity (H_E_) were calculated using Genepop version 4.2 (available at www.genepop.curtin.edu.au). In addition, a Mantel test was employed in Arlequin 3.0 to determine if there was a significant correlation between the population pairwise genetic distances (F_ST_/(1 − F_ST_)) and geographical distances of the localities studied.

Three different levels of individual analyses were separately performed for the cluster analysis: (i) 651 individuals from 33 populations based on all 24 microsatellite loci, (ii) 547 individuals from the western Sichuan Basin based on 13 rearranged loci, and (iii) 547 individuals from the western Sichuan Basin based on 11 normal loci. Individual-based Bayesian clustering assignment analyses were carried out using STRUCTURE version 2.3.4 (Pritchard et al., 2000). All analyses were run with a 1,000 burn-in period and 10,000 MCMC chains, with an admixture model with correlated allele frequencies. A variety of K values (1–10) were run to determine the profile of genetic clustering in the samples. The best-fit K value was estimated according to the maximum ΔK in STRUCTURE HARVESTER v0.6.93 (Earl and vonHoldt, 2012). We also separately ran a principal component analysis (PCA) for the three different levels of individuals using PCAGEN 1.2.1 software (Goudet, 1999). As PCAGEN and STRUCTURE rely on different assumptions, convergence in clustering should increase the reliability of our results. Furthermore, genetic bottleneck effects in populations were investigated with the software BOTTLENECK 1.2.02 (Piry et al., 1999), assuming a two-phase mutation model (TPM) with the settings recommended by authors for microsatellite data (95% single-step mutations with variance among multiple steps = 12).

To identify genetic differentiation on the CRs, it was necessary to assign rearrangement-linked markers to the reference genome. We chose a closely related species of *Q. boulengeri*, *Nanorana parkeri*, and mapped these loci to its whole-genome sequence (Sun et al., 2015), as well as to *Pyxicephalus adspersus*, which has a known chromosome-level assembly (Denton et al., 2018). As some markers have been identified *via* transcriptome sequencing (Xia et al., 2018), we anchored these markers to the transcript sequence and then to the reference genome. The alignments were performed using BLASTN v2.3, and we only retained the best hit with an e-value < 1e−20. In cases of multiple matches, we chose the one that was at least five orders of magnitude lower than that of the next best hit.

## Results

### Painting FISH of Different Karyotypes

**Figure 2:**
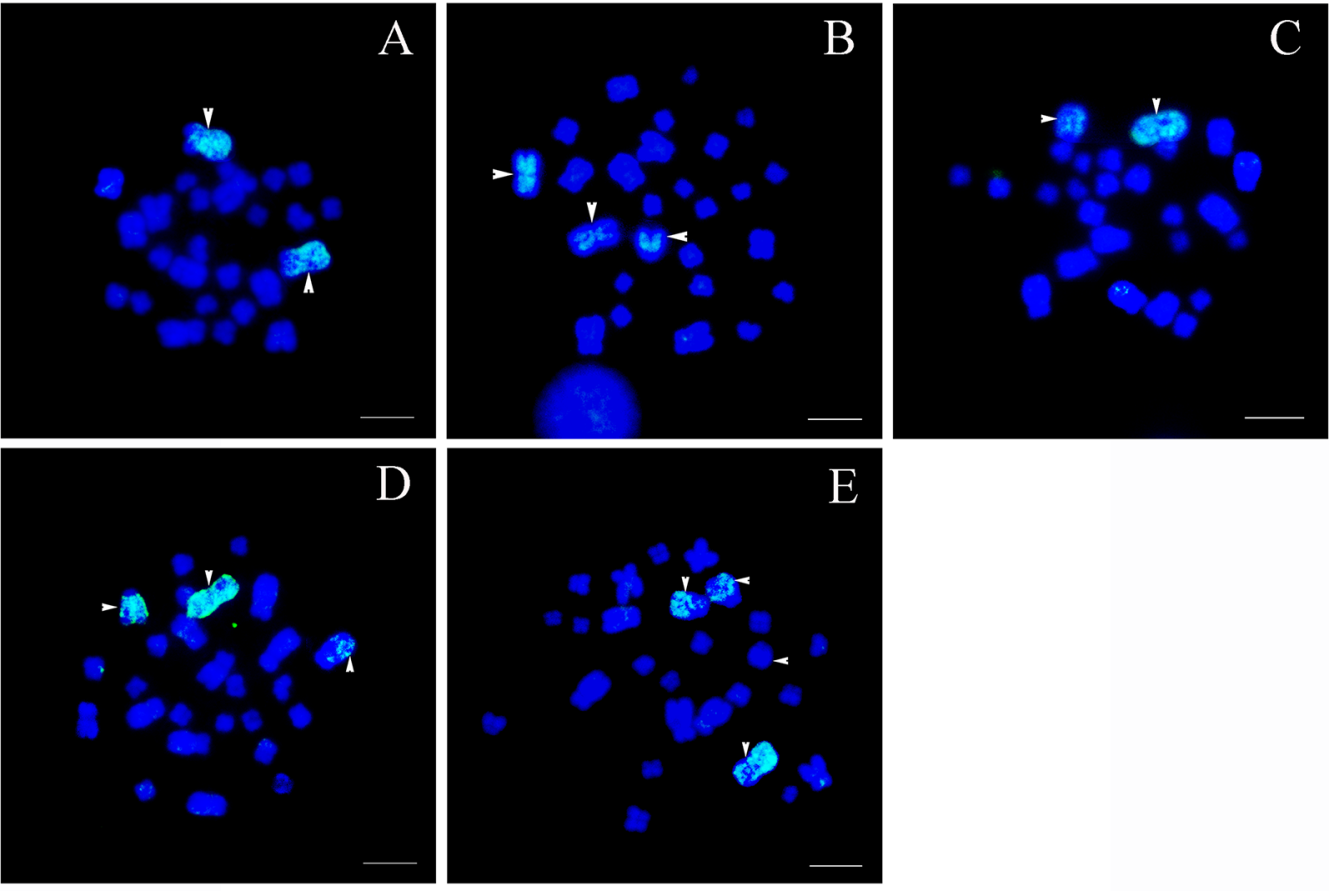
Chromosomal location of specific chromosome 1 probes, scale bar = 5 μm. **(A)** Type I: the fluorescent signals were detected only in the largest pair of homomorphic chromosome 1s (white arrows). **(B)** Type II: bright signals were found on a pair of homomorphic chromosome 1s and the long arm of sub-telocentric chromosome 6 (white arrows). **(C)** Type III: hybridization signals were located on the largest metacentric chromosome 1 and a telocentric chromosome including part of chromosome 1 (white arrows). **(D)** Type IV: fluorescent signals were observed in the largest metacentric chromosome 1, the telocentric chromosome including part of chromosome 1, and a large sub-telocentric chromosome 6 (white arrows). **(E)** Type V: hybridization signals were identified on the largest chromosome 1, the telocentric chromosome including part of chromosome 1, and a pair of large subtelocentric homomorphic chromosome 6s, while signals on one of the subtelocentric chromosome 6 were very faint (white arrows).

In all five karyotypes, strong and specific fluorescent signals were located on chromosome 1 and its translocated segments (**Figure 2**).

**Type I:** The normal karyotype. This karyotype contained a pair of homomorphic chromosome 1s and a pair of homomorphic chromosome 6s (MM/mm). The fluorescent signals were detected only in the largest pair of homomorphic chromosome 1 (MM). No signal was found on other chromosomes (**Figure 2A**).

**Type II:** The rearranged karyotype. This karyotype contained a pair of homomorphic chromosome 1s and a pair of heteromorphic chromosome 6s (MM/mSt). The fluorescent signals covered the whole pair of homomorphic chromosome 1 (MM) and the long arm of sub-telocentric chromosome 6 (St); the latter was the short arm of chromosome 1 translocated to chromosome 6 (**Figure 2B**).

**Type III:** The rearranged karyotype. This karyotype contained a pair of heteromorphic chromosome 1s and a pair of normal homomorphic chromosome 6s (MT/mm). Fluorescent signals were distributed in the largest metacentric chromosome 1 (M) and a telocentric chromosome (T); the latter included part of chromosome 1 (**Figure 2C**).

**Type IV:** The rearranged karyotype. This karyotype was characterized by translocation heterozygotes; both pairs of chromosome 1 and 6 were heteromorphic chromosomes (MT/mSt). Fluorescent signals were observed on the largest metacentric chromosome 1 (M), the telocentric chromosome including part of chromosome 1 (T), and a large sub-telocentric chromosome 6 (St) (**Figure 2D**).

**Type V:** The rearranged karyotype. This karyotype contained a pair of heteromorphic chromosome 1s and homologous chromosomal segments (MT/StSt). Fluorescent signals appeared on the largest chromosome 1 (M), the telocentric chromosome including part of chromosome 1 (T), and a pair of large subtelocentric homomorphic chromosome 6s (StSt), whereas signals on one of the subtelocentric chromosome 6s were rare and weak (**Figure 2E**). The fact that there were very few signals on other chromosomes may be attributable to the presence of repetitive sequences.

### High-Throughput Sequencing of Microdissected Chromosome 1

To sequence microdissected chromosome 1, a total of 6.29 million raw PE reads (PE250) were obtained from Illumina HiSeq. The raw reads have been deposited in the NCBI SRA database (Bioproject accession number: PRJNA493207). After removing adaptors and low-quality reads, the clean reads (4.58 million) were used for subsequent assembly. After assembly, we obtained 891,683 contigs with an average length of 301 bp, and there were more than 30,000 contigs longer than 500 bp. These contigs were used to design primers for nuclear gene sequence fragments and microsatellite loci.

### Genetic Differentiation Between Rearranged and Non-Rearranged Areas

The genetic differentiation index between rearranged and non-rearranged geographical areas of the 13 rearranged microsatellite loci (F_CT-R_) was 0.15 (*p* < 0.001), and the F_CT-N_ was 0.11 (*p* < 0.001) for the 11 normal microsatellite loci. This means that rearranged and non-rearranged areas had differentiated both in normal chromosomes and CRs. The Mantel test also revealed a significant correlation between geographical distances and genetic distances for all pairs of populations, including rearranged and non-rearranged ones (r = 0.492, *p* < 0.01).

**Figure 3:**
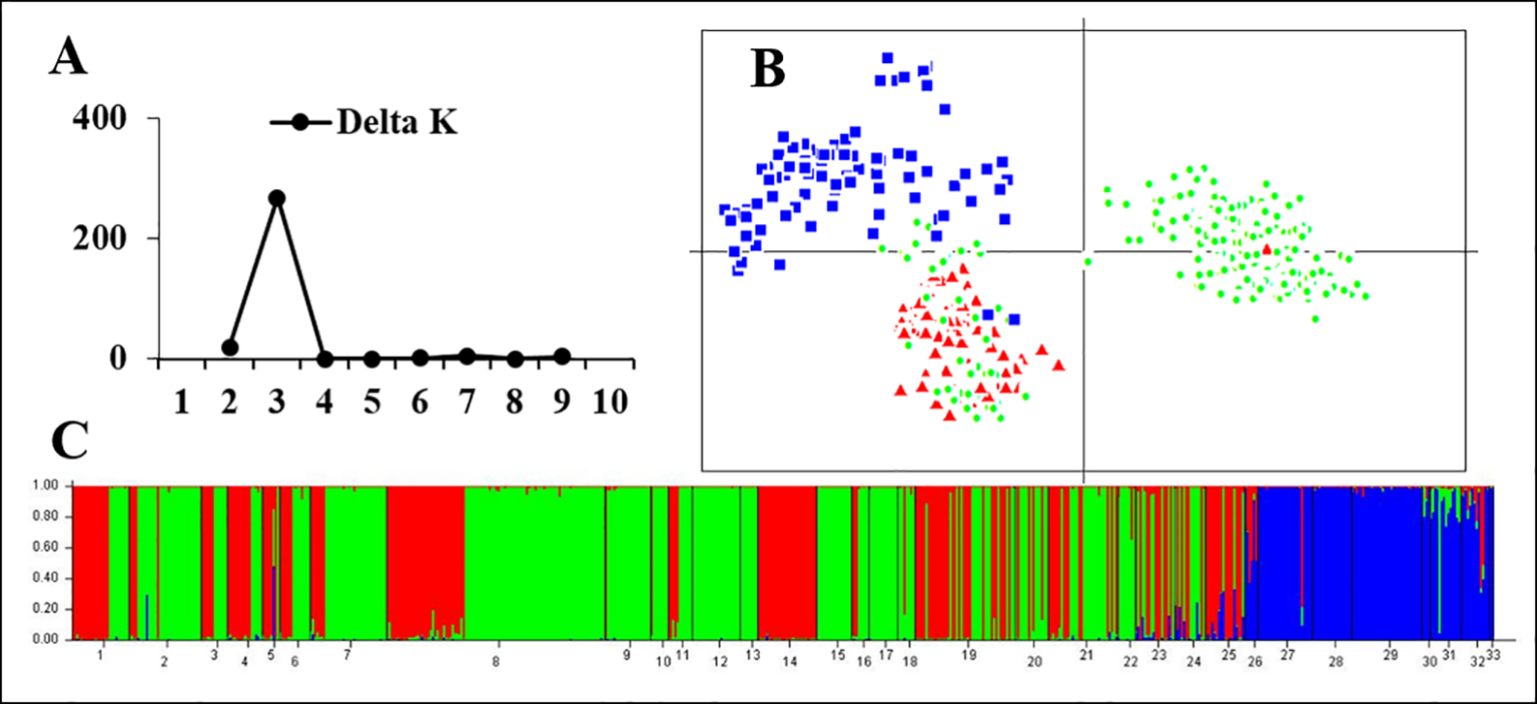
Genetic structure based on all 24 microsatellite loci for all samples in 33 populations. **(A)** The maximum delta K value was found for K = 3. **(B)** PCAGEN plots; blue square: individuals of non-rearranged areas; red triangle: individuals with rearranged karyotypes; green circle: individuals with the normal karyotype in the western Sichuan Basin. **(C)** Bayesian clustering, assignments of individuals were consistent with PCA results.

STRUCTURE and PCA analyses gave accordant results indicating significant genetic differences between rearranged and non-rearranged areas. In STRUCTURE analysis, the maximum ΔK was found for K = 3, and all individuals separated into three distinct groups. All individuals from non-rearranged areas belonged to blue clusters (**Figure 3**). Individuals from rearranged areas were clustered into two groups mainly according to karyotype. Rearranged karyotypic individuals were divided into the red cluster, with only one exception. Surprisingly, normal karyotypic individuals were assigned to either green or red clusters, with a large majority in the former. PCA analysis yielded consistent results: individuals were clustered into three groups corresponding to the STRUCTURE analysis.

Based on all 24 microsatellite loci, heterozygosity tests on most populations did not indicate recent bottlenecks under TPM (**Table S3**). There were no calculations for two populations (30GZLS and 33CQYY) because there were too few individuals. Comparing the observed heterozygosity (He) with the expected heterozygosity at neutral equilibrium (Heq), only one population (3PZCF) demonstrated significant heterozygosity excess (*p* < 0.01). Most of the populations displayed a normal L-shaped distribution, according to MODE-SHIFT tests. However, shifted modes were detected in populations 3PZCF, 16QLSK, and 20QLHJ, which may have been related to the low number of individuals in these populations (Piry et al., 1999).

### Genetic Differentiation Between Rearranged and Normal Individuals in the Western Sichuan Basin

In the western Sichuan Basin, the genetic differentiation index between rearranged and normal individuals of 13 rearranged microsatellite loci (F_ST-R_) was 0.20 (*p* < 0.001), whereas the F_ST-N_ was 0.07 (*p* < 0.001) for 11 normal microsatellite loci. This indicates that recombination was suppressed in rearranged loci but not in normal loci.

**Figure 4:**
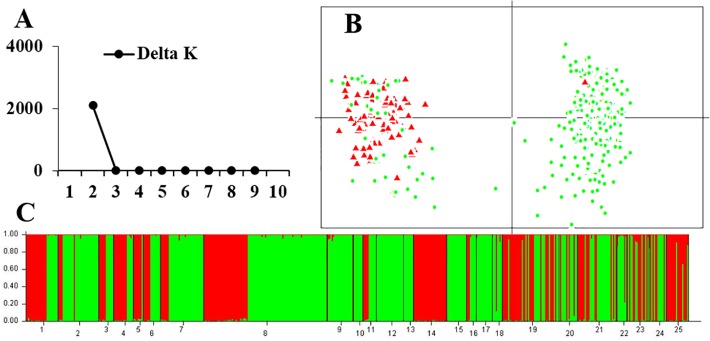
Genetic structure based on 13 rearranged microsatellite loci in 25 populations of the western Sichuan Basin. **(A)** The maximum delta K value was found for K = 2. **(B)** PCAGEN plots, red triangle: individuals with rearranged karyotypes; green circle: individuals with normal karyotype. **(C)** Bayesian clustering, individuals were assigned to the respective clusters mainly according to karyotype.

**Figure 5:**
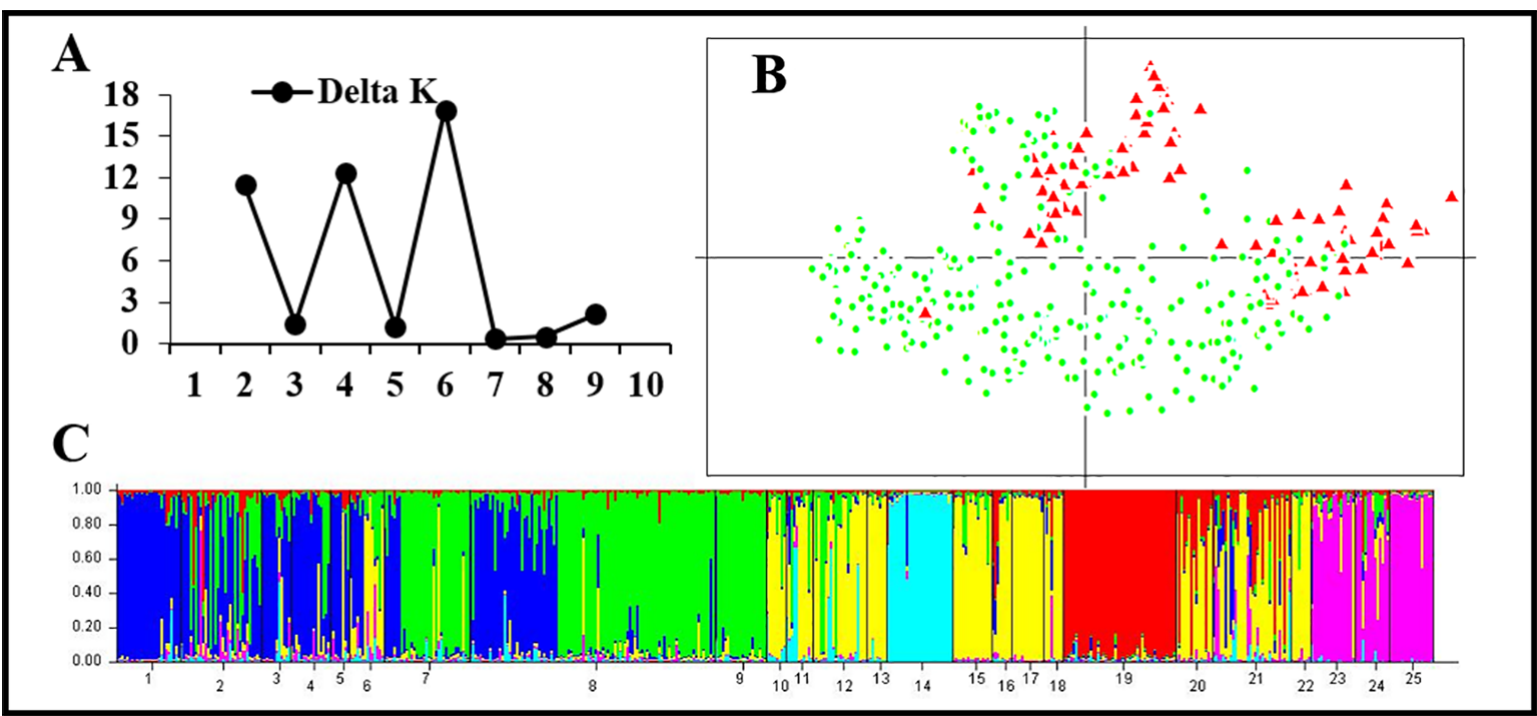
Genetic structure based on 11 normal microsatellite loci in 25 populations of the western Sichuan Basin. **(A)** The maximum delta K value was found for K = 6. **(B)** PCAGEN plots, red triangle: individuals with rearranged karyotypes; green circle: individuals with normal karyotype. **(C)** Bayesian clustering, assignments of individuals were independent of karyotype.

For the 13 rearranged microsatellite loci, both STRUCTURE and PCA analyses demonstrated two well-separated groups. The maximum ΔK of STRUCTURE analysis was found at K = 2, and individual assignments were the same as the results shown in **Figure 4**. The majority of rearranged karyotypic individuals were divided into the red cluster, and normal karyotypic individuals belonged to either the green or red clusters. This distribution means that chromosome rearrangements significantly affected genetic recombination and differentiation. A partial Mantel test for 13 rearranged microsatellite loci in the western Sichuan Basin was performed, and the results revealed a significant correlation between geographical distances and genetic distances for all population pairs (r = 0.349, *p* < 0.01). Based on 11 normal microsatellite loci, however, STRUCTURE analysis resulted in chaotic clusters, and PCA analysis demonstrated that individuals appeared to be randomly distributed within one single cluster (**Figure 5**). In addition, genetic distances were significantly correlated with geographic distances based on Mantel tests of 11 normal microsatellite loci (r = 0.347, *p* < 0.01).

**Figure 6:**
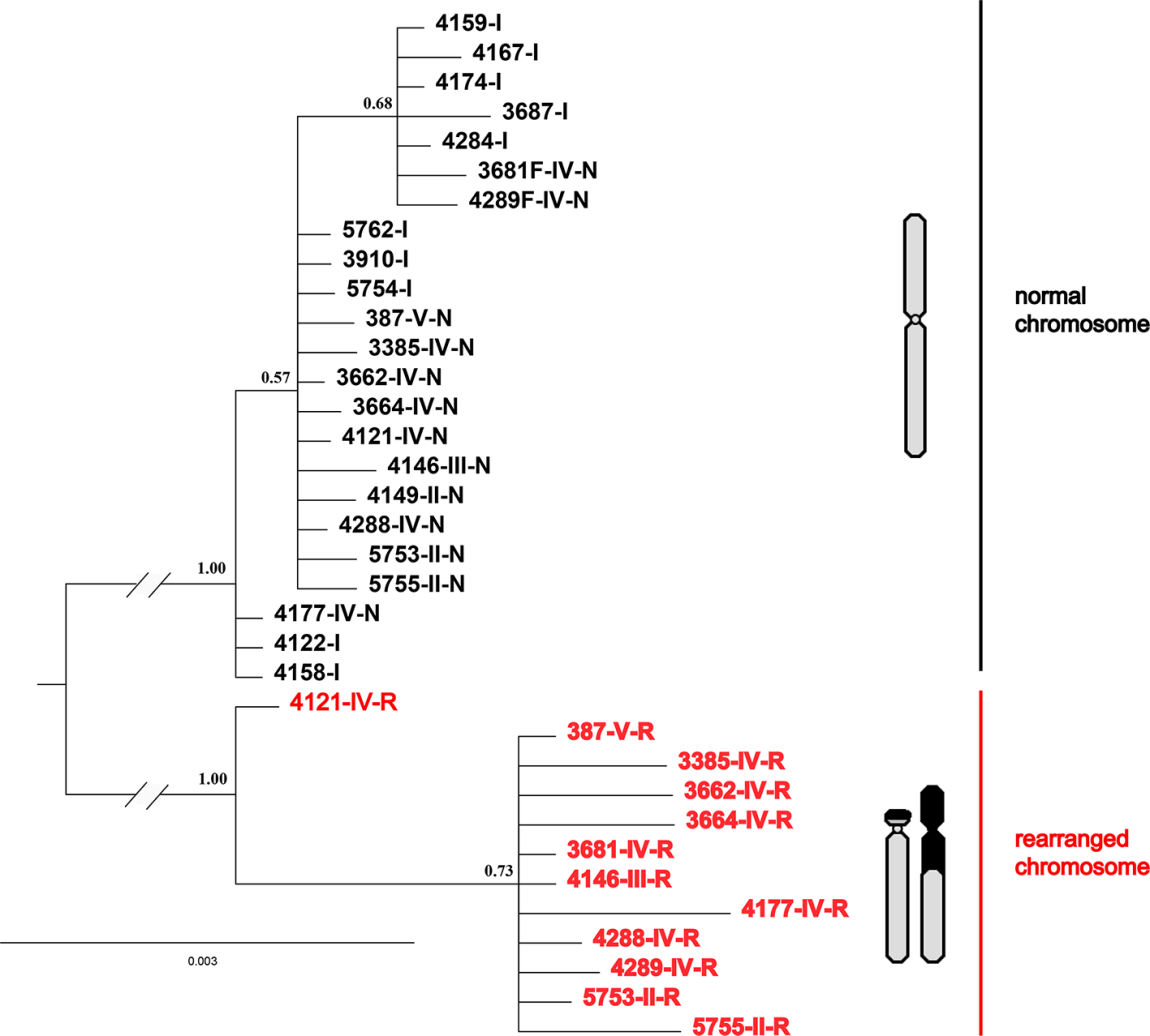
Phylogenetic tree of *Q. boulengeri* inferred from Bayesian analysis of four nuclear gene fragments located on rearranged chromosomes. Numbers beside nodes are Bayesian posterior probabilities supporting the phylogram displayed.

Interestingly, all four nuclear fragments located on rearranged chromosomes (QBR-B11, QBR-C27, QBR-C7, and QBR-D60) presented two distinctly divided alleles in rearranged karyotypic individuals, and only had one allele in non-rearranged individuals. These sequences were deposited in GenBank under the accession numbers MH990684–MH990787. The data set for the four nuclear fragments had 2,440 nucleotide sites, and Bayesian phylogenetic reconstruction demonstrated a topology with two distinct clades with strong node support (**Figure 6**). Sequences in one of the clusters (red cluster) were all from rearranged karyotypic individuals, suggesting that this cluster corresponded to rearrangement-specific haplotypes and rearranged chromosomes. The remaining cluster contained sequences both of rearranged and normal individuals, and may represent normal haplotypes in different karyotypic individuals. These two well-supported clades may correspond to a CR and normal chromosome, respectively (**Figure 6**). These results were supported by network analysis of nuclear loci (**Figure S1**), alleles of rearranged chromosome and normal chromosome are separated distinctly for all four nuclear markers. These results also indicate strongly suppressed recombination between translocated and normal chromosomes.

The results of bottleneck detection are presented in **Table S3**. Most of the populations have not undergone significant demographic bottlenecks. For the 13 rearranged loci, two populations (3PZCF and 4WCYX) demonstrated significant heterozygosity excess (*p* < 0.01), and no populations displayed heterozygosity excess based on the 11 normal loci. Shifted modes were only detected in populations 3PZCF, 16QLSK, and 20QLHJ based on 13 rearranged loci, the same as the results using all 24 loci. For 11 normal loci, population 18QLGH replaced 20QLHJ with a shifted mode. The rest of the populations in the western Sichuan Basin showed a normal L-shaped distribution.

### Phylogenetic Analysis Based on Mitochondrial Fragments

**Figure 7:**
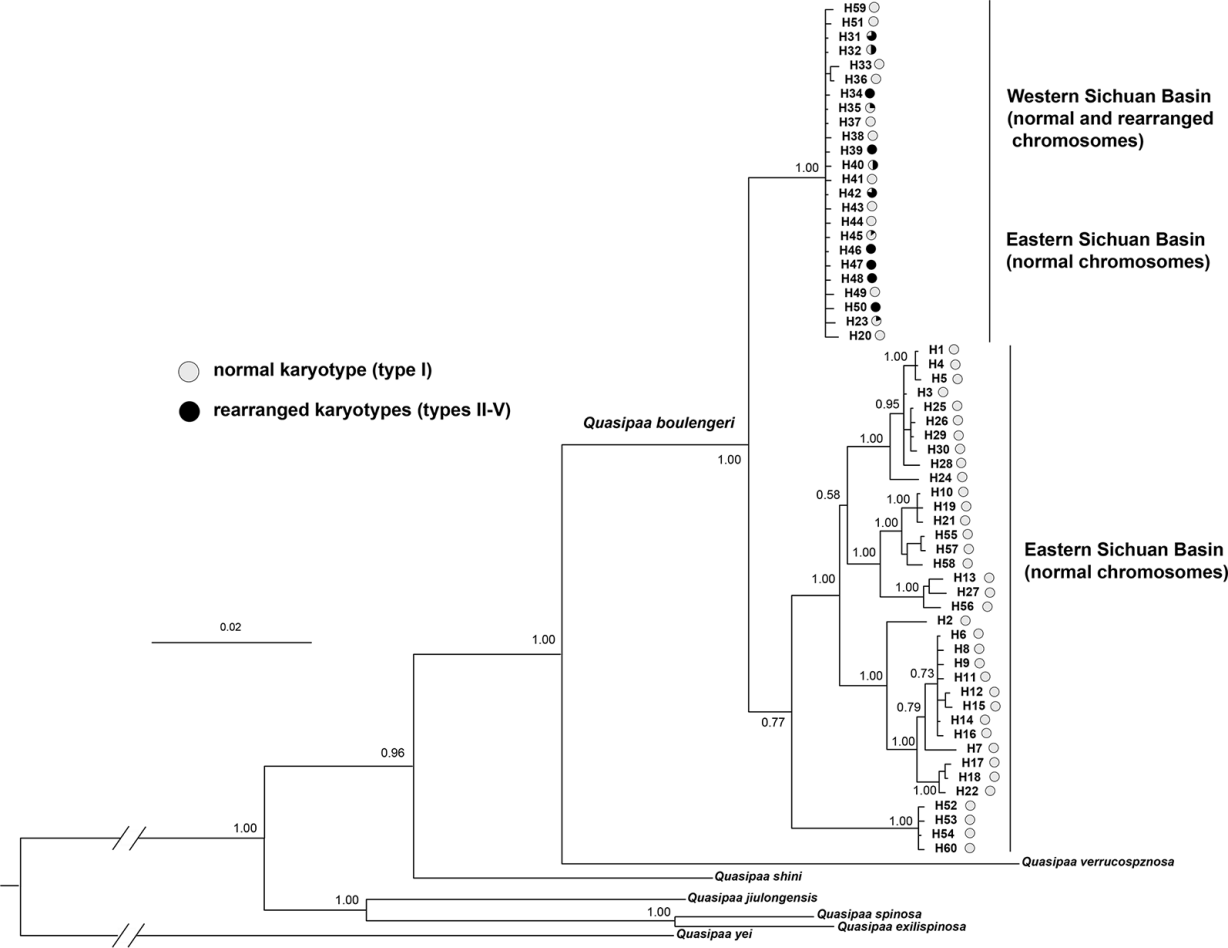
Bayesian phylogenetic reconstruction using the *cox1* + *cytb* + *12s* + *16s* data set. The number at each node is the Bayesian posterior probability supporting the phylogram displayed.

The *cox1 + cytb + 12s + 16s* data set had 60 haplotypes and 2,363 nucleotide sites (**Table S1**). The 50% majority consensus tree is illustrated in **Figure 7**. There is strong support for monophyly of all *Q. boulengeri* sequences included in the analysis. Within *Q. boulengeri*, individuals from non-rearranged areas appeared in two clades. Individuals from the western Sichuan Bain, including rearranged and normal karyotypes, were clustered into the same clade, and this clade also included several individuals from the eastern group with normal karyotypes (**Figure 7**). Several haplotypes were shared by individuals with normal and translocated karyotypes (**Figure 7**, **Table S1**), demonstrating that CRs did not affect the mitochondrial fragments.

### Range of Suppressed Recombination

The rearrangement-linked markers, which showed significant genetic differentiation, were mapped to the whole genomes of *N. parkeri* and *P. adspersus*, respectively. Most of these makers received a unique alignment, and each aligned locus anchored to a specific scaffold in *N. parkeri* (**Table S4**). Intriguingly, two thirds (4/6) of rearrangement-linked loci were mapped to chromosome 2 in *P. adspersus*, and non-rearranged loci were mapped to other chromosomes (**Table S4**). According to the aligned position on chromosome 2 (2.5–73.6 Mb with a total of 174 Mb) of *P. adspersus*, it seems that recombination is suppressed over a large region on rearranged chromosomes, which occupy almost half of the whole chromosome. This assumption was also supported by the alignment to *N. parkeri*, as each locus anchored to a specific scaffold (scaffold N50s for *N. parkeri* was 1.05 Mb).

## Discussion

### Single Origin of Chromosomal Translocation Polymorphisms

*Quasipaa boulengeri* is widely distributed in low mountainous regions along the edges of the Sichuan Basin and nearby areas in southern China. Individuals with translocated karyotypes are only found in populations in the western part of the Basin, with inter- and intra-population karyotype variations (Qing et al., 2012) (**Figure 1**). According to FISH of 5S rDNA and measurement of chromosomal lengths, it has been assumed that the five karyomorphs were caused by a reciprocal translocation mutation and combination of unbalanced gametes (Qing et al., 2012). However, this hypothesis lacks support, as FISH of 5S rDNA only informs about the homology between normal chromosome 1 and translocated chromosome 1, not the homology between normal chromosome 1 and translocated chromosome 6. Moreover, the high degree of condensation of amphibian chromosomes in metaphase may cause large measurement errors in the relative length and centromeric indices of chromosomes (Schmid, 1978; Schempp and Schmid, 1981). Comparatively, whole-chromosome painting is more sensitive, refined, and definitive.

The single origin of the translocation polymorphisms was supported by the WCP evidence and genetic structure clustering. If the origin were a single translocation event, the pattern of chromosomes involved in rearrangement would be the same, and vice versa. Moreover, all arrangements of translocation segments would be in high concordance with each other, regardless of karyotype or population. Considering that the translocation exchange involved almost the whole short arm of chromosome 1 and a very small chromosome segment from the long arm of chromosome 6 (Qing et al., 2012), the WCP results indicate overall homology between chromosome 1 (M) and translocated chromosomes 1 (T) and 6 (St) from different populations (**Figure 2**). In addition, both STRUCTURE and PCA analyses divided rearranged karyotypic individuals into the same cluster based on 13 rearranged microsatellite loci (**Figure 4**). These results were supported by phylogenetic analysis of four nuclear fragments located on rearranged chromosomes (**Figure 6**), which showed that the rearrangement-specific haplotypes and CRs belonged to the same cluster. These results coincide with the prediction that translocation independently evolved just once in this species, that is, a single ancient individual carrying a translocation heterozygote led to the formation of the five karyomorphs.

### Suppressed Recombination-Driven Genetic Evolution of Chromosomal Reciprocal Translocation

The different clustering results between rearranged and non-rearranged microsatellite loci strongly support the “recombination suppression” model in translocated chromosomes. In the western Sichuan Basin, the genetic differentiation between rearranged and normal individuals of rearranged microsatellite loci (F_ST-R_) was much higher than that of normal microsatellite loci (F_ST-N_). This indicates suppressed recombination in the rearranged loci but not in the normal loci. This was supported by the STRUCTURE and PCA analyses of rearranged (**Figure 4**) and normal (**Figure 5**) microsatellite loci, and phylogenetic analysis of rearranged nuclear fragments (**Figure 6**) and mitochondrial fragments (**Figure 7**). Recent models have demonstrated that CRs can suppress recombination, but most of the theoretical and empirical emphasis to date has been on inversions (Noor et al., 2001; Kirkpatrick, 2010; Guerrero et al., 2012). Our results indicate that gene flow can be reduced by suppressing recombination in the case of reciprocal translocation.

Although the fitness of the hybridized karyotypes in *Q. boulengeri* was reduced, this cannot be the reason for genetic differentiation into a balanced chromosomal polymorphism. Theoretically, nine kinds of karyologically different progeny could be produced by the four gametes, but four of the anticipated karyotypes were not found in any populations of this spiny frog (Qing et al., 2012). Additionally, three hybridized karyotypes (types II, III, and V) were very rare (**Table S1**). Generally, chromosomes normally segregate during meiosis in a translocation homozygote, according to Mendelian principles. In *Q. boulengeri*, however, the translocation homozygote (Type IX) is absent. These absences and rarities could be the result of lethal effects and genetic imbalances (duplication or deficiency) that are harmful to the organism or its progeny (Roberts, 1976). It seems that the heterozygosity of reciprocal translocation leads to a reduction in hybrid fertility due to aberrant meiosis, germ cell death, and embryonic lethality (Searle, 1993). These effects reduce gene flow globally over the genome and cause genetic differentiation (Garagna et al., 2014). However, without the translocation homozygote, chromosomal polymorphisms in *Q. boulengeri* could be in a floating or balanced state. Genetic differentiation was only found in translocated chromosomes (**Figures 4**, **6**), not in normal chromosomes or the mitochondrial genome (**Figures 5**, **7**). If the genetic differentiation were caused by the loss of recombinant progeny, it would exist not only in translocated chromosomes but also in the whole genome. Further, the rare, unbalanced karyotypes (types II, III, and V) were also only differentiated in translocated chromosomes (**Figures 4** **–** **7**). Indeed, for translocation heterozygotes, the patterns of chromosome segregation during meiosis would produce genetically unbalanced gametes that cause semisterility. But such hybrid breakdown cannot lead to speciation in this species because of the absence of the translocation homozygote. The observed genetic differentiation related to rearrangement was only in the translocated chromosomes, and is better explained by suppressed recombination between translocated chromosomes rather than hybrid unfitness.

Suppressed recombination plays an important role in genetic differentiation *via* inversion and Robertsonian fusion, but its role in reciprocal translocation is questionable. The suppressed recombination model proposes that gene exchange between two chromosomes is reduced or suppressed near rearrangements (Rieseberg, 2001; Navarro and Barton, 2003a). This model is broadly supported by theoretical and empirical studies on various species with inversion (Hoffmann and Rieseberg, 2008; Kirkpatrick, 2010) and a study on shrews with Robertsonian fusions, where restricted gene flow was generally larger in CRs than across common chromosomes (Basset et al., 2006). However, several studies of translocation in house mice have challenged this view, arguing for a predominance of hybrid unfitness over suppressed recombination in limiting gene flow between chromosomal races (Franchini et al., 2010; Hauffe et al., 2011; Giménez et al., 2013). The centromeric regions of some non-rearranged chromosomes in the house mouse also show genetic differentiation between hybridizing groups, indicating a complex interplay (such as selective sweeps and/or epistasis) between chromosomal rearrangements and other parts of the genome (Förster et al., 2016). In *Q. boulengeri*, the distinct differences in gene flow between rearranged and normal chromosomes support the suppressed recombination model.

Genetic differentiation on translocated chromosomes was found (**Table S4**) not only near the breaking point of rearrangement, suggesting that the differentiation between karyotype groups of spiny frog is due to a complex mix of factors. Following the spread and/or fixation of a CR, the suppressed recombination region could be extended by accumulating repetitive sequences or capturing alleles that are adapted to the local environment (Kirkpatrick, 2017; Potter et al., 2017). Such adaptations or selection would also be associated with suppressed recombination (Charlesworth, 2016; Kirkpatrick, 2017). Adaptive genes are suggested to partially reside in large CR regions, as their reduced recombination promotes adaptive divergence (Barth et al., 2017). Recently, striking geographic variation in the differentiation of sex chromosomes and sex determination based on sex-linked markers has been found in *Q. boulengeri* (Yuan et al., 2018). Coincidentally, the area with differentiated sex chromosomes occurs in the inter- and intra-population karyotype variation. From this study, it was clear that suppressed recombination existed broadly in the rearranged loci (**Figures 4** **–** **7**). Such recombination suppression may drive adaptive evolution by bringing together advantageous gene combinations (Navarro and Barton, 2003b; Hoffmann and Rieseberg, 2008). Although CRs place organisms and their progeny at a selective disadvantage, strong selection favors locally adapted alleles in populations connected by migration (Kirkpatrick and Barton, 2006; Kirkpatrick, 2017). If the translocation captures two or more alleles that are adapted to the local environmental conditions and provides a selective advantage, it can spread. In *Q. boulengeri*, sex-linked markers are located on chromosome 1, which suggests that when the translocation first appeared, it captured preexisting sex-linkage alleles that were locally adapted (Yuan et al., 2018). This indicates that reduced recombination and local adaptation could drive hetero-karyotype advantage in specific demes (Kirkpatrick, 2017).

In conclusion, recombination suppression would facilitate genetic divergence in species with reciprocal translocation. Very few natural polymorphisms involving inter-chromosomal reciprocal translocations are known in animal populations. To test the driving force of genetic differentiation, it often needs to seek the early stages of chromosome differentiation. However, such early stages were not easy to be found, as the chromosomal race caused by rearrangement can be fixed in even 500 years (Britton-Davidian et al., 2000). In *Q. boulengeri*, there was no geographic isolation or reproductive isolation between western and eastern populations, indicating it represented early stages of chromosome differentiation. Without the translocation homozygote (type IX), chromosomal polymorphisms in *Q. boulengeri* could be in a floating or balanced state, and our study provided evidence of genetic differentiation driven by reciprocal translocation. Balanced chromosomal polymorphisms is a good model to test the driving mechanism of population genetic differentiation, because it is difficult to investigate the mechanism of genetic differentiation in the case of deeply divided chromosomal races. The region of suppressed recombination occurred between rearranged chromosomes and its homologous chromosome, may related to other functional differentiation, such as varied sex-linked marker differentiation in the different populations of *Q. boulengeri* (Yuan et al., 2018).

## Data Availability Statement

The datasets supporting this article have been uploaded as part of the **Supplementary Material**. The sequences of frogs in this study were deposited in GenBank with accession numbers MH990684–MH990787. Microdissected chromosome 1 sequencing data were deposited in NCBI SRA with accession no. SRR7904274.

## Ethics Statement

All animal care and experimental procedures were approved by the Chengdu Institute of Biology Animal Care and Use Committee [CIB2015003].

## Author Contributions

YX and XZ designed the study. XY, WL, and SY performed the experiments. YX, XY, WL, and SY analyzed the data. XY and YX wrote the paper. XZ improved the paper.

## Funding

This study was supported by the National Key Programme of Research and Development, Ministry of Science and Technology (2017YFC0505202), and the National Natural Science Foundation of China (NSFC-31772439, NSFC-31572241), the Youth Innovation Promotion Association of CAS (2019362).

## Conflict of Interest

The authors declare that the research was conducted in the absence of any commercial or financial relationships that could be construed as a potential conflict of interest.
