# Supplementary material for: The Origin and Evolution of Chromosomal Reciprocal Translocation in Quasipaa boulengeri (Anura, Dicroglossidae)
Source: Front Genet. 2020 Jan 21;10:1364. doi: 10.3389/fgene.2019.01364 (PMC6985567; doi:10.3389/fgene.2019.01364)
Supplement: Supplementary file 1 [file Image_1.pdf]

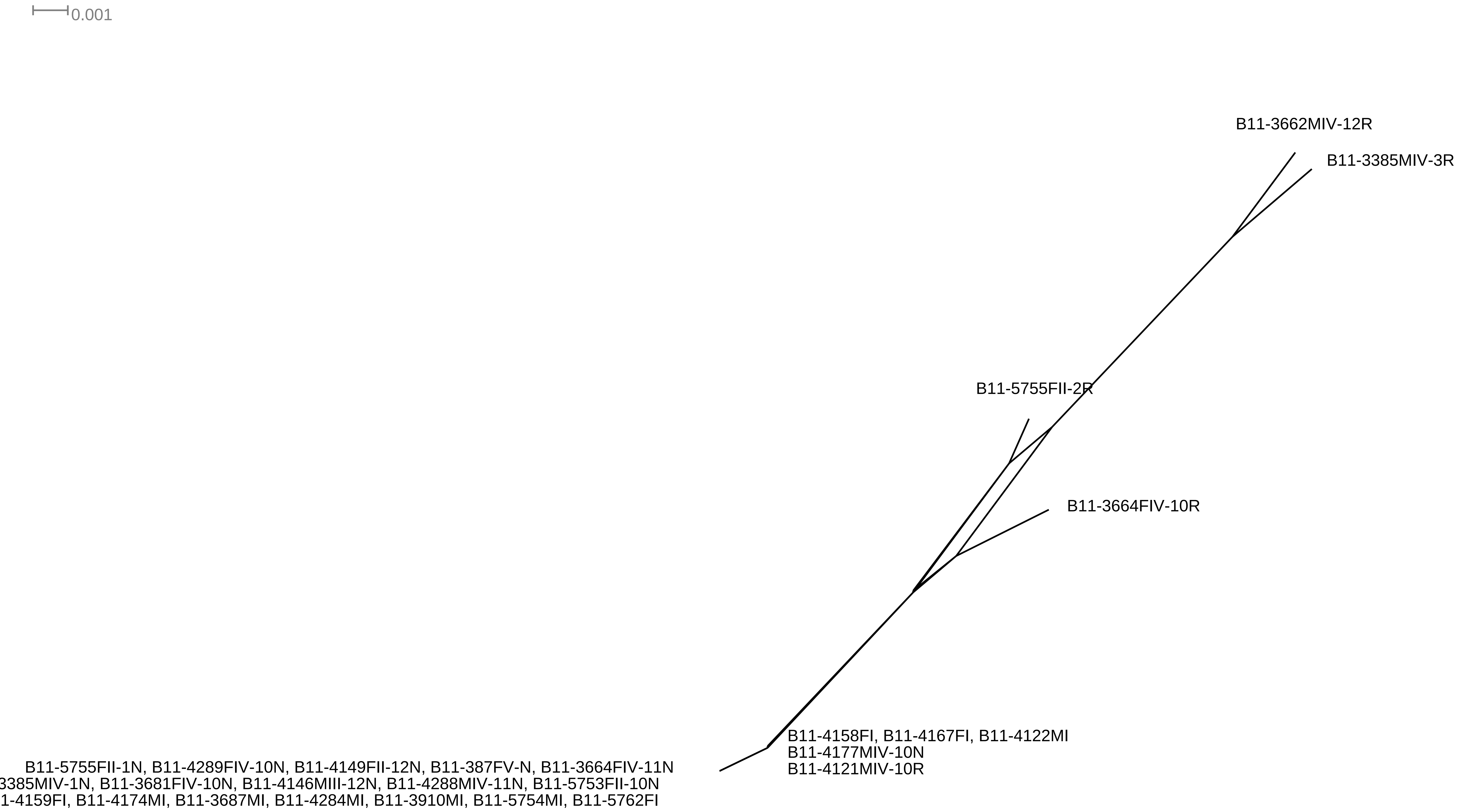
0.001
B11-3662MIV-12R
B11-3385MIV-3R
B11-5755FII-2R
B11-3664FIV-10R
B11-4158FI, B11-4167FI, B11-4122MI
B11-4177MIV-10N
B11-4121MIV-10R
B11-5755FII-1N, B11-4289FIV-10N, B11-4149FII-12N, B11-387FV-N, B11-3664FIV-11N
3385MIV-1N, B11-3681FIV-10N, B11-4146MIII-12N, B11-4288MIV-11N, B11-5753FII-10N
1-4159FI, B11-4174MI, B11-3687MI, B11-4284MI, B11-3910MI, B11-5754MI, B11-5762FI

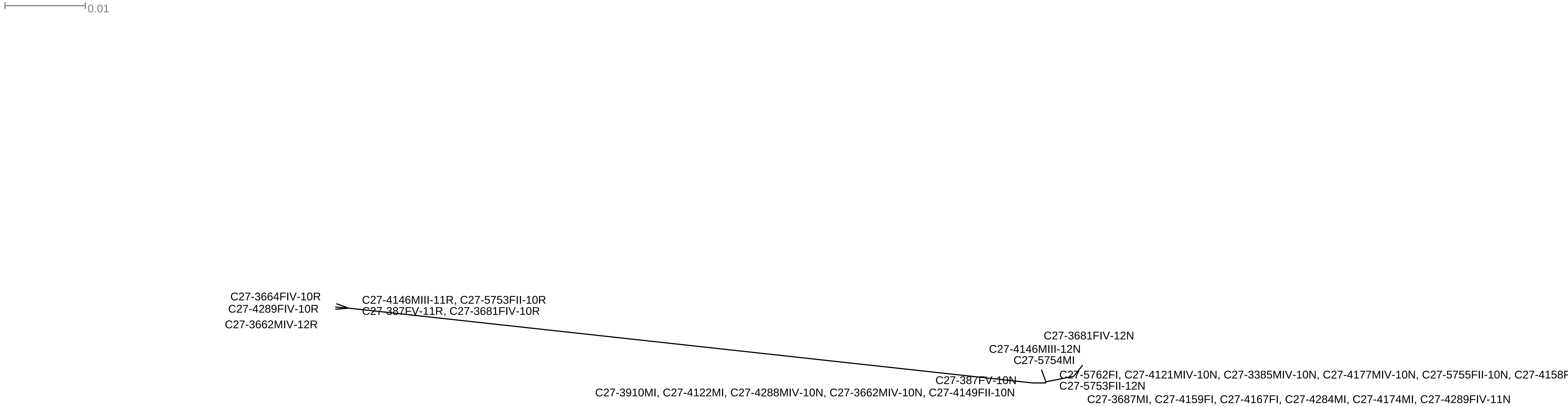
0.01
C27-3664FIV-10R
C27-4289FIV-10R
C27-3662MIV-12R
C27-4146MIII-11R, C27-5753FII-10R
C27-387FV-11R, C27-3681FIV-10R
C27-3681FIV-12N
C27-4146MIII-12N
C27-5754MI
C27-5762FI, C27-4121MIV-10N, C27-3385MIV-10N, C27-4177MIV-10N, C27-5755FII-10N, C27-4158F
C27-387FV-10N
C27-5753FII-12N
C27-3910MI, C27-4122MI, C27-4288MIV-10N, C27-3662MIV-10N, C27-4149FII-10N
C27-3687MI, C27-4159FI, C27-4167FI, C27-4284MI, C27-4174MI, C27-4289FIV-11N

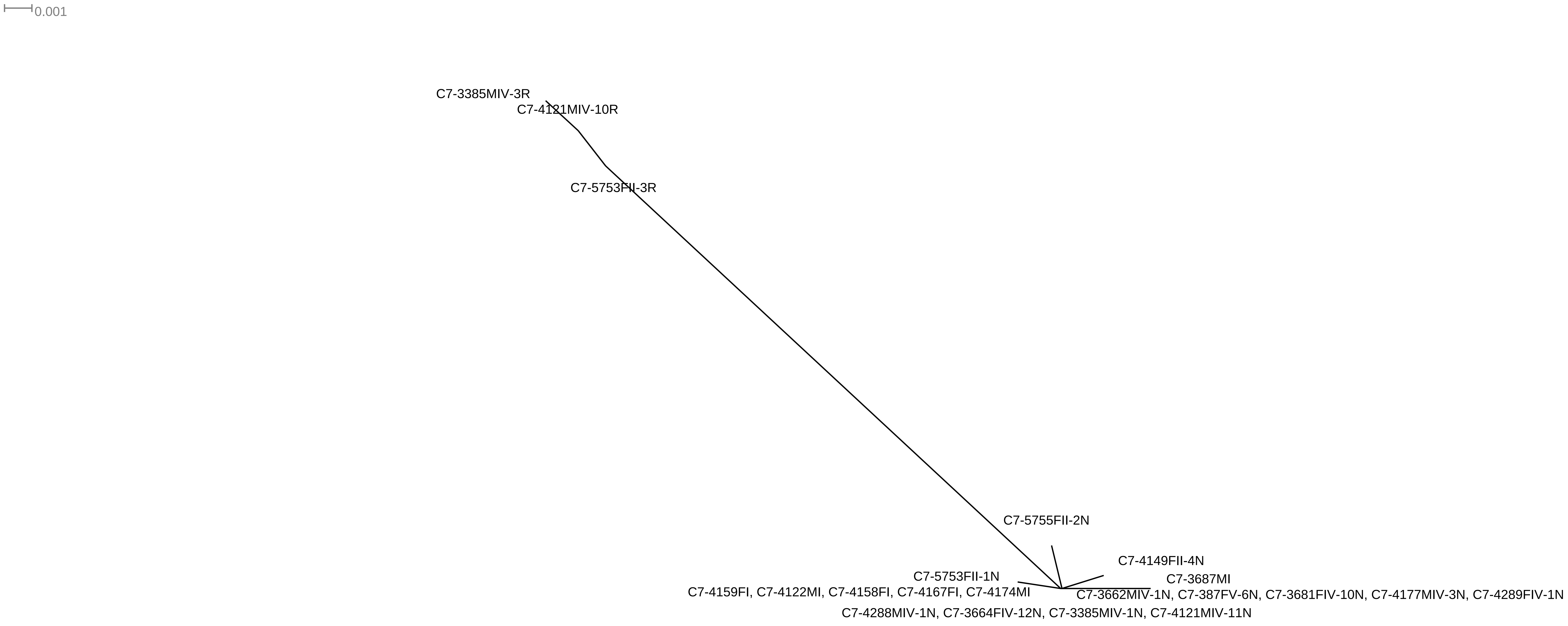
0.001
C7-3385MIV-3R
C7-4121MIV-10R
C7-5753FII-3R
C7-5755FII-2N
C7-4149FII-4N
C7-5753FII-1N
C7-3687MI
C7-4159FI, C7-4122MI, C7-4158FI, C7-4167FI, C7-4174MI
C7-3662MIV-1N, C7-387FV-6N, C7-3681FIV-10N, C7-4177MIV-3N, C7-4289FIV-1N
C7-4288MIV-1N, C7-3664FIV-12N, C7-3385MIV-1N, C7-4121MIV-11N

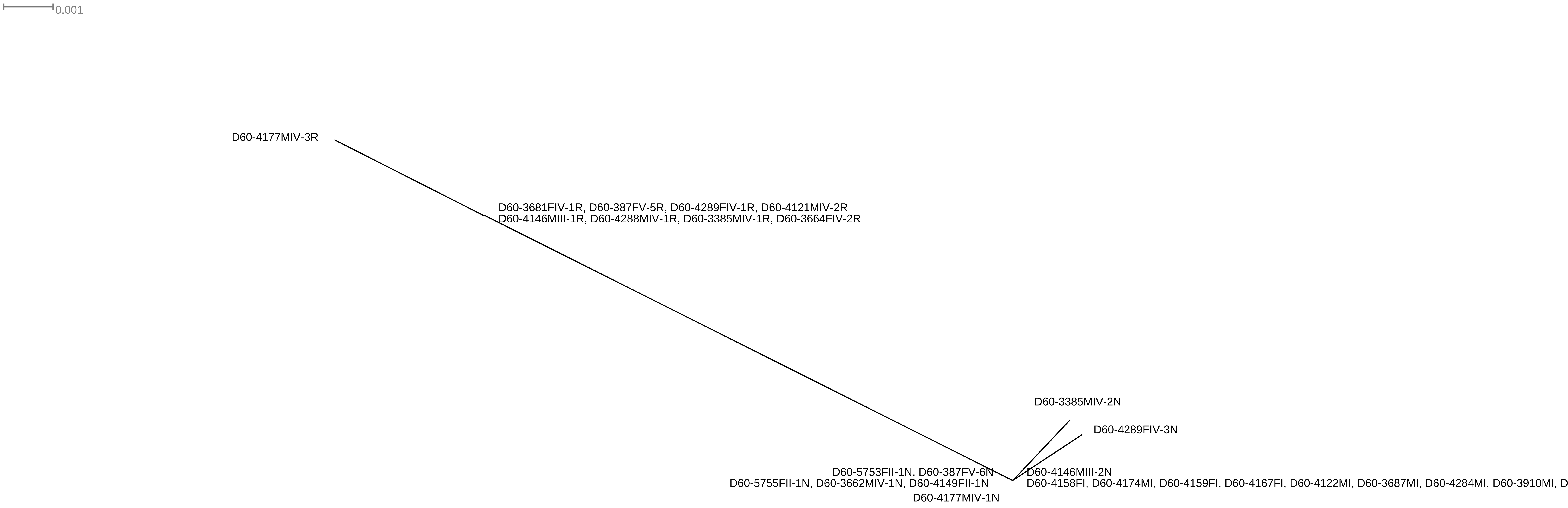
0.001
D60-4177MIV-3R
D60-3681FIV-1R, D60-387FV-5R, D60-4289FIV-1R, D60-4121MIV-2R
D60-4146MIII-1R, D60-4288MIV-1R, D60-3385MIV-1R, D60-3664FIV-2R
D60-3385MIV-2N
D60-4289FIV-3N
D60-5753FII-1N, D60-387FV-6N
D60-5755FII-1N, D60-3662MIV-1N, D60-4149FII-1N
D60-4177MIV-1N
D60-4146MIII-2N
D60-4158FI, D60-4174MI, D60-4159FI, D60-4167FI, D60-4122MI, D60-3687MI, D60-4284MI, D60-3910MI, D
