# Supplementary material for: The Origin and Evolution of Chromosomal Reciprocal Translocation in Quasipaa boulengeri (Anura, Dicroglossidae)
Source: Front Genet. 2020 Jan 21;10:1364. doi: 10.3389/fgene.2019.01364 (PMC6985567; doi:10.3389/fgene.2019.01364)
Supplement: Supplementary file 3 [file Table_2.docx]

**Table S2** Primer details for the 24 microsatellite loci and four nuclear gene fragments of the rearranged chromosome.

| **Locus** | **Primers (5’-3’; for/rev)** | **Repeat Motifs** | **Tm( °C)** | **Location** |
| --- | --- | --- | --- | --- |
| X | GCCCGCTCTATTCCTCATTTCC ACTCCGATCCCAGCCTACAG | (ATC)_5_ | 57 | rearranged chromosome |
| Y15 | ACGATTCCACTACCCTCTG GGATCTGGTTGCTTTCAATAG | (ATCC)_5_ | 56 | rearranged chromosome |
| D68 | GCATCCCTAACAATTTCCCAC CCCCGTAATTTATCAGTGACAGT | (AGC)_5_ | 55 | rearranged chromosome |
| C27 | GAGAGAAGACAGCCGACCCT GCCTCGTGTGCTGGGTCTG | (CTC)_7_-(CGC)_3_ | 56 | rearranged chromosome |
| D12 | GAGGCAATAGCAAAGCTGGAC GGACCAGATCAGTAAGGGGAG | (CAG)_7_ | 55 | rearranged chromosome |
| QB28 | TACATCAGACAATCATGGCTGC  TGCCTTCTCTGACAGAACACTG | (AGAT)_14_ | 55 | rearranged chromosome |
| D34 | GCCTTGGGATGCTCTGTCATCT  GCGTCAGCATAGCCGGTCCT | (TG)_6_-(GCT)_4_ | 56 | rearranged chromosome |
| D60 | CTTCTCCCTCCTTTGTAACCT  AGTATAAATCCAGCCGAGGGTCC | (TG)_7_ | 56 | rearranged chromosome |
| B08 | GTCCCATTCAGATAGAACA  TACATACAAGTAGGGGGCTC | (GT)_9_ | 56 | rearranged chromosome |
| B11 | GTTGCCTACCTTTCCATAGA  GAACCTCTCATAGCCTCCCA | (AT)_6_ | 55 | rearranged chromosome |
| QBc7 | TACAGAACAATCCA AAGGCT  GTCAATGGTGGTCAAAGTCA | (TCTA)_12_ | 60 | rearranged chromosome |
| QBy3 | AAGCCCTGTAGAAATTGTTA  AAGCAGACT AAGCAGGTGG | (ATAC)_10_ | 60 | rearranged chromosome |
| QBb42 | AGCCTGGATT GTAAGTACCT  CGTAGAAACCT AGAATGTAGC | (ATCT)_17_ | 60 | rearranged chromosome |
| QB15 | AACAGGGATACACATGCTTCAA  ATGCCGAGGTAGGAGAGTACAG | (GTGA)_13_ | 55 | normal chromosome |
| QB12 | CTAAATCCAGGTGGTGACCTTT  AGTGGAGCCCACTGTAGTGATT | (TAGA)_14_ | 55 | normal chromosome |
| QB02 | AGCTTTCTGCTCATTTCCTGTC  GAAGACAACCAAAGTCCCTGAG | (ACTAT)_9_ | 55 | normal chromosome |
| D56 | CTTCAGTCCGGTTCTAGCTC  AGGACTTCACACACTGGATGC | (GCT)_5_ | 64 | normal chromosome |
| B12 | CTGCACCCTTACCACCACA  GCCTCCATTTCAGTGTAGGT | (AT)_7_ | 56 | normal chromosome |
| N | ATCCCTTTTAAGGAACTCCAG  CAGACAAGCGCAACGGTAAGG | (TGT)_5_ | 58 | normal chromosome |
| QBb26 | TGGAGTGGCACAGTTTTGATAGT  TAATTCGTTACTTCTGCTAAACCAT | (AGAT)_24_ | 60 | normal chromosome |
| QBb1 | CCTTACATTGGTTGTCCTCGTC  AAGAGTGTCGCTGGGATCAGG | (TCTA)_23_ | 60 | normal chromosome |
| QBb5 | CTGCTTGCCTTTGTGTAAT  ATCTCCAGGATAAAGTTGTG | (TCTA)_11_ | 60 | normal chromosome |
| QBb45 | GGGTTAGGTAAGGATAAGAGAT  ATCAGTAGGCAGCAGACATAC | (GATA)_17_ | 58 | normal chromosome |
| QBz16 | TATTCATTCACATCAATCCCTG  CCTCTCTAAATCAGCCCTAGTATG | (ACAT)_9_ | 60 | normal chromosome |
| QBR-B11 | AGACTGGAGGAAATGGTGTC  GCATGATGGTAATTGTAGCG | --- | 55 | rearranged chromosome |
| QBR-C7 | AACACATCAGGAGGCACACA  GATTAAACATGGTCCAAACC | --- | 60 | rearranged chromosome |
| QBR-C27 | ATAATACGAGTCCCCAGACA  ATTTGTAACTTCCGCTCTGT | --- | 55 | rearranged chromosome |
| QBR-D60 | TCATACTCAATAACTGTGCA  GATAGGTAAGCTGTCTCTCC | --- | 55 | rearranged chromosome |
