# Supplementary material for: The Origin and Evolution of Chromosomal Reciprocal Translocation in Quasipaa boulengeri (Anura, Dicroglossidae)
Source: Front Genet. 2020 Jan 21;10:1364. doi: 10.3389/fgene.2019.01364 (PMC6985567; doi:10.3389/fgene.2019.01364)
Supplement: Supplementary file 4 [file Table_3.docx]

**Table S3**. The results of BOTTLENECK test for each population.

| Population | N-all (*P* value) | Mode-shift | N-R (*P* value) | Mode-shift | N-N (*P* value) | Mode-shift |
| --- | --- | --- | --- | --- | --- | --- |
| 1LMS | 17 (0.046)* | L | 10 (0.072) | L | 7 (0.323) | L |
| 2DJYHK | 9 (0.055) | L | 4 (0.072) | L | 5 (0.332) | L |
| 3PZCF | 21 (0.001)** | S | 12 (0.003)** | S | 9 (0.070) | S |
| 4WCYX | 16 (0.179) | L | 13 (0.000)** | L | 4 (0.135) | L |
| 5WCXK | 16 (0.336) | L | 11 (0.016)* | L | 4 (0.141) | L |
| 6QCS | 17 (0.110) | L | 11 (0.024)* | L | 6 (0.508) | L |
| 7JXHP | 10 (0.104) | L | 5 (0.147) | L | 5 (0.309) | L |
| 8YEC | 14 (0.485) | L | 8 (0.479) | L | 6 (0.584) | L |
| 9DYWS | 8 (0.028)* | L | 3 (0.026)* | L | 5 (0.309) | L |
| 10DYXL | 9 (0.111) | L | 4 (0.087) | L | 3 (0.113) | L |
| 11DYHM | 13 (0.433) | L | 8 (0.513) | L | 6 (0.528) | L |
| 12DYXY | 13 (0.474) | L | 5 (0.344) | L | 8 (0.236) | L |
| 13DYXC | 11 (0.491) | L | 7 (0.361) | L | 3 (0.124) | L |
| 14GTS | 14 (0.516) | L | 9 (0.245) | L | 5 (0.330) | L |
| 15QLDT | 7 (0.048)* | L | 4 (0.272) | L | 3 (0.074) | L |
| 16QLSK | 15 (0.354) | S | 7 (0.584) | S | 7 (0.474) | S |
| 17QLNB | 9 (0.213) | L | 5 (0.509) | L | 4 (0.184) | L |
| 18QLGH | 10 (0.099) | L | 3 (0.014)* | L | 7 (0.478) | S |
| 19QLDZ | 17 (0.058) | L | 10 (0.079) | L | 7 (0.332) | L |
| 20QLHJ | 9 (0.038)* | S | 4 (0.053) | S | 5 (0.289) | L |
| 21TTS | 14 (0.519) | L | 10 (0.111) | L | 4 (0.137) | L |
| 22YABFX | 9 (0.274) | L | 4 (0.218) | L | 5 (0.587) | L |
| 23EMPX | 13 (0.472) | L | 9 (0.266) | L | 4 (0.135) | L |
| 24EMS | 13 (0.411) | L | 8 (0.510) | L | 5 (0.278) | L |
| 25EMLM | 14 (0.550) | L | 8 (0.500) | L | 6 (0.523) | L |
| 26YBPS | 15 (0.290) | L | 10 (0.142) | L | 5 (0.444) | L |
| 27GSKX | 10 (0.174) | L | 5 (0.193) | L | 5 (0.409) | L |
| 28SXLY | 10 (0.172) | L | 5 (0.233) | L | 5 (0.402) | L |
| 29KKS | 13 (0.425) | L | 9 (0.290) | L | 4 (0.114) | L |
| 30GZLS | - | - | - | - | - | - |
| 31HNXFS | 14 (0.459) | L | 6 (0.383) | L | 7 (0.363) | L |
| 32HBYC | 13 (0.382) | L | 6 (0.261) | L | 7 (0.506) | L |
| 33CQYY | - | - | - | - | - | - |

N-all (*P* value): the number of loci with heterozygosity excess for all 24 loci; N-R (*P* value): the number of loci with heterozygosity excess for 13 rearranged loci; N-N (*P* value): the number of loci with heterozygosity excess for 11 normal loci. *: *P* < 0.05; **: *P* < 0.01.
