# Supplementary material for: The Origin and Evolution of Chromosomal Reciprocal Translocation in Quasipaa boulengeri (Anura, Dicroglossidae)
Source: Front Genet. 2020 Jan 21;10:1364. doi: 10.3389/fgene.2019.01364 (PMC6985567; doi:10.3389/fgene.2019.01364)
Supplement: Supplementary file 5 [file Table_4.docx]

Table S4 Chromosome-wide distribution of rearrangement linked markers, by mapping 24 microsatellite loci onto the genome of *Nanorana parkeri* and *Pyxicephalus adspersus*.

| Locus | *Nanorana parkeri* | *Pyxicephalus adspersus*  (aligned position) |
| --- | --- | --- |
| X* | Scaffold607 | **Chr 2** (69875530-69531965) |
| Y15* | --- | --- |
| D68* | Scaffold2917 | **Chr 2** (2577971-2582290) |
| C27* | Scaffold3213 | **Chr 2** (8097082-8096910) |
| D12* | Scaffold124 | Chr 3 |
| QB28* | Scaffold9 | --- |
| D34* | Scaffold261 | **Chr 2** (73560842-73659555) |
| D60* | Scaffold34 | Chr 6 |
| B08* | Scaffold1345 | --- |
| B11* | Scaffold1234 | --- |
| QBc7* | --- | --- |
| QBy3* | Scaffold508 | --- |
| QBb42* | Scaffold263 | --- |
| QB15** | Scaffold1684 | --- |
| QB12** | Scaffold804 | --- |
| QB02** | Scaffold1002 | --- |
| D56** | Scaffold154 | Chr 4 |
| B12** | Scaffold8530 | Chr 4 |
| N** | Scaffold120 | --- |
| QBb26** | --- | --- |
| QBb1** | Scaffold166 | Chr 11 |
| QBb5** | Scaffold220 | Chr 10 |
| QBb45** | --- | --- |
| QBz16** | Scaffold1489 | --- |

Note: “---” means not aligned to reference genome. “*” means the locus is located on rearranged chromosome; “**” means the locus is located on normal chromosome.
